# Effect of Remimazolam on Pain Perception and Opioid-Induced Hyperalgesia in Patients Undergoing Laparoscopic Urologic Surgery—A Prospective, Randomized, Controlled Study

**DOI:** 10.3390/medicina60010123

**Published:** 2024-01-09

**Authors:** Cheol Lee, Junsung Lim, Hansol Hong, Hyungjong Yu, Hayoung Lee

**Affiliations:** 1 Department of Anesthesiology and Pain Medicine, Wonkwang University School of Medicine Hospital, 895 Muwang-ro, Iksan 54538, Republic of Korea; sy4957@naver.com (J.L.); gksthf686@gmail.com (H.H.); afirmacion7@naver.com (H.Y.); 2 Department of Nursing, Wonkwang University School of Medicine Hospital, 895 Muwang-ro, Iksan 54538, Republic of Korea

**Keywords:** analgesia, desflurane, hyperalgesia, pain threshold, pain perception, remifentanil, remimazolam

## Abstract

*Background and Objectives*: The effects of midazolam, a benzodiazepine, on pain perception are complex on both spinal and supraspinal levels. It is not yet known whether remimazolam clinically attenuates or worsens pain. The present study investigated the effect of intraoperative remimazolam on opioid-induced hyperalgesia (OIH) in patients undergoing general anesthesia. *Materials and Methods*: The patients were randomized into three groups: group RHR (6 mg/kg/h initial dose followed by 1 mg/kg/h remimazolam and 0.3 μg /kg/min remifentanil), group DHR (desflurane and 0.3 μg /kg/min remifentanil) or group DLR (desflurane and 0.05 µg/kg /min remifentanil). The primary outcome was a mechanical hyperalgesia threshold, while secondary outcomes included an area of hyperalgesia and clinically relevant pain outcomes. *Results*: Group RHR had a higher mechanical hyperalgesia threshold, a smaller hyperalgesia postoperative area at 24 h, a longer time to first rescue analgesia (*p* = 0.04), lower cumulative PCA volume containing morphine postoperatively consumed for 24 h (*p* < 0.01), and lower pain intensity for 12 h than group DHR (*p* < 0.001). However, there was no significant difference in OIH between groups RHR and DLR. *Conclusions*: Group RHR, which received remimazolam, attenuated OIH, including mechanically evoked pain and some clinically relevant pain outcomes caused by a high dose of remifentanil. Further research is essential to determine how clinically meaningful and important the small differences observed between the two groups are.

## 1. Introduction

Opioid-induced hyperalgesia (OIH) is a condition in which the long-term use of high doses of opioids results in pain sensitization. Paradoxically, a painful stimulus with the same intensity may result in even more intense pain. OIH can cause delays in discharge and discomfort due to higher pain scores and increased use of analgesics and their related effects [1]. Remifentanil is a type of ultra-short-acting μ opioid receptor agonist that is commonly used in general anesthesia. It has a fast onset and short half-life of elimination and does not accumulate in the body over time. Remifentanil is metabolized by non-specific esterases found in plasma and tissues and has a constant context-sensitive half-life. However, using high doses of remifentanil during surgery may cause acute pain and hyperalgesia after the procedure, resulting in poor early postoperative analgesia outcomes [2].

Pharmacological interventions are available to prevent opioid-induced hyperalgesia (OIH), which include a variety of drugs that target different mechanisms. NMDA receptor antagonists, such as ketamine and magnesium sulfate, act on NMDA receptors. COX inhibitors, such as parecoxib and ketorolac, as well as GABA analogs, such as gabapentin and pregabalin, have also been explored. Other potential treatments for OIH include adenosine, dexmedetomidine (an α2-adrenergic receptor agonist), and anesthetics such as propofol and nitrous oxide. However, it is not fully understood how effective these treatments are in attenuating OIH, highlighting the complexity of managing this condition and the need for individualized treatment strategies [1,2].

The effects of midazolam, a benzodiazepine, on the perception of pain, are complex at both a spinal and supraspinal level. Midazolam primarily works as an analgesic at the spinal level, indicating its potential effectiveness for pain relief. This effect is due to its interaction with GABA receptors, which increases inhibitory neurotransmission and reduces pain signals [3,4]. Conversely, midazolam’s effects at the supraspinal level in the brain may increase pain perception by altering pain processing pathways and neuronal sensitization in the central nervous system [5,6]. Remimazolam, like other benzodiazepines, is lipophilic, which allows it to cross biological membranes, including the blood-brain barrier. Given this property, it is likely that remimazolam can penetrate into the cerebrospinal fluid (CSF) to some extent after entering the central nervous system [7,8]. However, the extent to which remimazolam reduces pain and its concentration in plasma compared to CSF should be confirmed in specific pharmacokinetic studies. In addition, it is not yet known whether remimazolam clinically attenuates or worsens pain.

Therefore, we hypothesized that intraoperative remimazolam would enhance or attenuate pain perception during high doses of remifentanil under general anesthesia. The present study investigated the effect of intraoperative remimazolam on hyperalgesia induced by high doses of remifentanil in terms of mechanically evoked pain and clinically relevant pain outcomes. The study focused on patients who underwent single-port laparoscopically assisted urologic surgery.

## 2. Materials and Methods

### 2.1. Study Design

A study with randomized and controlled methods was conducted at Wonkwang University Hospital. This study was approved by the University’s Institutional Review Board (IRB #2023-001-003) and written informed consent was obtained from all subjects participating in the trial. The trial was registered prior to patient enrollment at clinicaltrials.gov (NCT05866315, Principal investigator: Cheol Lee, Date of registration: 28 March 2023). https://clinicaltrials.gov/study/NCT05866315 (accessed on 6 October 2023)

### 2.2. Participants

The inclusion criteria were:

1. Patients aged 20 to 65 years;
2. Those undergoing single-port laparoscopically assisted urological surgery;
3. Patients classified as ASA I–III.

The exclusion criteria were:

1. Known allergies to remimazolam or remifentanil;
2. History of alcohol or drug abuse, psychiatric disorders;
3. Presence of acute cardiovascular diseases;
4. Refractory hypertension;
5. Other respiratory or neuromuscular diseases;
6. Chronic pain or treatment with opioid-containing analgesics within 24 h before surgery;
7. Patients who were unable to understand the PCA device or who had contraindications to self-administration of opioids.

### 2.3. Randomization and Procedure

A computer-generated table was used to randomly assign patients to one of three groups, using a block size of 2. Patients were informed that they had an equal chance of being assigned to any of the groups. The first group, RHR, received an initial dose of 6 mg/kg/h of remimazolam, followed by 1 mg/kg/h and 0.3 µg/kg/min of remifentanil. The second group, DHR, received 1 minimum alveolar concentration (MAC) of desflurane, adjusted by 1 vol% titration to maintain mean arterial pressure (MAP) and bispectral index (BIS) levels, and 0.3 µg/kg/min of remifentanil. The third group, DLR, received desflurane at a dose determined by 1 MAC, adjusted by 1 vol% titration to maintain MAP and BIS levels, and 0.05µg/kg/min of remifentanil.

Patients were blindly assigned to groups, and two attending anesthesiologists conducted the anesthetic procedures and evaluated outcomes. One attending anesthesiologist performed the anesthesia induction following the study protocol. The other attending anesthesiologist, who was unaware of the assigned groups, measured all outcomes throughout the perioperative period.

### 2.4. Anesthesia and Perioperative Care

For self-administering pain relief, patients were instructed to use a visual analog scale (VAS) and a patient-controlled analgesia (PCA) device prior to their surgery. They were advised to use the device whenever they felt pain. No premedication was given before the patients arrived in the operating room. During their stay, the patients’ pulse oximetry, automated cuffed blood pressure (BP), electrocardiogram (ECG), and end-tidal CO_2_ (ETCO_2_) levels were regularly monitored. As required for routine management, arterial and urinary catheters were also used.

For fluid therapy, Crystalloid (Ringer’s solution) and 6% HES 130/0.4 were used in a balanced electrolyte solution (Volulyte) in all three groups. Allogeneic red blood cells were only given to those with hemoglobin levels of 8.0 g/dL or lower. The blood and fluid were heated using an infusion fluid-heating apparatus (FMS2000, Belmont Instrument Corporation, Billerica, MA, USA) in order to maintain a temperature of 37 °C. A forced-air warming blanket (Bair Hugger Blankets, Augustine Medical, Inc., Eden Prairie, MN, USA) was applied to the upper body except for the surgical site to deliver heat at 38 °C.

During surgery, the operating room was kept at a temperature of 20–22 °C with a relative humidity of 20–60%. To ensure patient comfort and safety, warmed intravenous and irrigating fluids were used, as well as heated and humidified peritoneal insufflation gas (CO_2_). The patient’s temperature was monitored using a tympanic thermometer before anesthesia and just before extubation. A nasopharyngeal temperature probe was then inserted through the nostril to a depth of 9.5 to 10 cm to monitor the core temperature every 10 min until the end of the surgery.

Anesthesia was induced with an intravenous bolus of remifentanil (1 µg/kg), followed by propofol (1–2 mg/kg), and rocuronium (0.9 mg/kg) was administered to facilitate intubation. As previously stated, the amount of remifentanil administered was consistent across all groups, and anesthesia was sustained using desflurane at an initial end-tidal concentration of 1 MAC along with a medical air-oxygen blend (with 50% oxygen fraction).

During the surgery, the amount of anesthesia given was gradually adjusted by increasing the desflurane concentration by 1 vol% based on changes in the patient’s heart rate and blood pressure. The goal was to maintain a BIS between 40–60. If the patient’s MAP dropped below 60 mmHg, it was considered low blood pressure, and the patient was given 250 mL of lactated Ringer’s solution. If this did not work, they were given 10 mg of ephedrine. If the patient’s heart rate (HR) was less than 50 beats per minute, it was considered slow, and they were given 0.5 mg of atropine. When the train-of-four (TOF) ratio returned to 25% after surgery, the muscle relaxant’s effects were reversed using pyridostigmine (0.2 mg/kg) and glycopyrrolate (0.008 mg/kg). Once the BIS values had reached 80 and the patient was able to breathe on their own, they were taken off the ventilator. After the final surgical stitch was placed, the remifentanil infusion was stopped.

Pain management was achieved with morphine (60 mg), ketorolac (180 mg), and ramosetron (0.6 mg) by PCA pump (Accufuser^®^ WooYoung Medical, Seoul, Korea). With a 15-min lockout time, the device was programmed to deliver a basal infusion of 0.5 mL/h and bolus doses of 2 mL. Patients were given intravenous ketorolac (30 mg) if they reported a visual analog scale (VAS) of 30 or higher in PACU. An additional 15 mg was administered if the patient continued to report a VAS of 30 or higher.

Upon arrival at the post-anesthesia care unit (PACU), infrared tympanic thermometers were used to measure the core body temperature three times. When a patient’s core body temperature dropped below 36 °C, a forced-air warming blanket delivered warm air at 42 °C. The warming blanket’s temperature was adjusted to 38 °C once the core body temperature reached 36 °C.

Post-anesthesia shivering (PAS) incidence and severity were assessed during the patient’s stay in the PACU. Shivering was rated on a bedside evaluation for PAS severity. The scale evaluated shivering on the masseter, neck, and chest wall and rated it as none (grade 0) if no shivering was noted, mild (grade 1) if shivering was localized to the neck and thorax, moderate (grade 2) if shivering involved upper extremity movements in addition to the neck and thorax, and severe (grade 3) if shivering involved movements of the trunk, and upper and lower extremities. In cases of persistent or intractable PAS, a 25 mg meperidine injection was administered.

The clinically relevant pain outcomes associated with OIH were defined as increased first rescue analgesia, pain intensity, or cumulatively consumed PCA volume containing morphine for 24 h after surgery. The mechanically evoked pain associated with OIH was defined as an increased area of hyperalgesia around the incision and decreased mechanical hyperalgesia threshold.

### 2.5. Mechanically Evoked Pain pPotocol

The methods used in this study to measure mechanically evoked pain are similar to those used in a previous study [9]. Prior to the surgery, we utilized Von Frey filaments (Bioseb™, Chaville, France) to measure the pain threshold for mechanical punctuate stimuli. Following the surgery, we conducted a repeat measurement 24 h later on both the dominant upper inner arm and peri-incisional areas. The Von Frey filament is composed of 20 monofilaments with the same length but different diameters. The numerical rating of the filaments (1.65–6.65) is said to correspond to a logarithmic function of forces ranging from 0.008–300 g, according to the manufacturer. As a fiber of a particular length and diameter is pressed against a testing area straight-on, the force applied grows as the researcher pushes forward with the probe, until the fiber eventually bends.

When the fiber bends, pushing the probe further will not add more force to the test area. However, it allows for the consistent application of forces within a broad range of tolerance on the tested surface. Continuously applying force for one second is followed by its removal. The patients were asked to answer with either “yes” (meaning they felt the stimulation) or “no” (meaning they did not feel the stimulation). If the patient did not detect the pressure, a larger filament was used to apply pressure of gradually increasing intensity until a response was observed. If a response was detected, the pressure was immediately increased using a larger filament. The minimum force (g/mm^2^) required to bend a von Frey filament that is perceived as painful was defined as the mechanical hyperalgesia threshold. To determine the mechanical hyperalgesia threshold, von Frey filaments were used on areas located 2 cm away from the umbilicus before the surgery or 2 cm from the incision site of the single port after the surgery. The filaments were applied at four points, both horizontally and vertically.

A von Frey filament (with a number of 6.1 and a force of 100 g) was used to stimulate the area of hyperalgesia around the surgical incision. The stimulation started outside the area of hyperalgesia where the patient did not experience any pain. It was then gradually moved toward the incision site until the patient reported a clear change in perception. To measure the area of hyperalgesia, tests were performed by moving along straight lines, creating quadrilaterals that were 5 cm away from the incision site, 24 h post operation. The data collected were then plotted on graph paper to determine the surface area. The point where the patient first experienced pain, discomfort, or a sharp sensation was marked. If there was no change in sensation, the test was halted 1 cm away from the incision.

#### Outcome Measures

The primary outcome was the mechanical hyperalgesia threshold. Secondary outcomes included the area of hyperalgesia around the surgical incision at 24 h post operation. The time to first rescue analgesia, total analgesic (ketorolac) consumption, pain intensity with VAS on movement at 1, 6, 12, 24, and 48 h post operation, cumulatively consumed PCA volume containing morphine for 24 h post operation, perioperative hypothermia, and PAS.

### 2.6. Sample Size and Statistical Analysis

Based on the results of the preliminary study, subjects in the three groups had mechanical hyperalgesia thresholds of 138, 120, and 149 g/mm^2^. The standard deviation (SD) between the groups was 35.5%. To show a noteworthy difference with a power of 80% and an α-coefficient of 0.05, it was necessary to have a sample size of 30 patients in each group. A final sample size of 36 patients per group was calculated based on a dropout rate of 20%. Data are expressed as the mean ± SD or percentage of patients.

We conducted a comparison between groups based on various factors such as age, body weight, duration of anesthesia, remifentanil dose administered, mechanical hyperalgesia threshold, area of hyperalgesia around the surgical incision, time to first rescue analgesia, pain intensity, and cumulatively consumed PCA volume containing morphine for 24 h after surgery. This was carried out using a one-way analysis of variance (ANOVA). A Bonferroni correction of the significance level was used for post-hoc comparisons. The categorical data, including sex, ASA classification, intraoperative hypothermia, hypothermia in the PACU, and PAS incidence and severity, were analyzed using the Chi-square test. In order to assess the relationship between two binary variables, we used the phi coefficient (rφ) as a measure of association. To analyze the association between a continuous variable and a dichotomous variable, we utilized the point-biserial correlation (rpb). A statistically significant difference was considered to be *p* < 0.05.

## 3. Results

**Figure 1:**
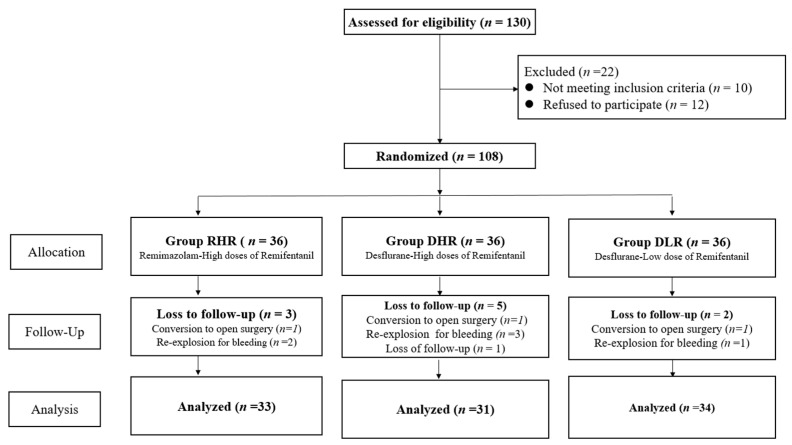
Consort flow diagram.

Of 130 assessed patients, 22 were excluded due to not meeting criteria or refusing to participate. Of the enrolled 108 patients, 10 were subsequently excluded due to various reasons such as conversion to open surgery, loss of follow-up, and re-exploration for postoperative bleeding, leaving 98 patients for analysis, as shown in the CONSORT flow diagram (Figure 1).

No significant differences in age, sex, height, body weight, ASA physical status, type of surgery, duration of anesthesia, duration of operation, total fluid administered, and the severity of PAS were observed between the three groups. The volume of desflurane used was significantly higher in group DLR than in group DHR (*p* < 0.01). The total dose of remifentanil administered was significantly lower in group DLR compared to the other two groups (*p* < 0.01). Hypotension was significantly higher in group DLR than in the other two groups (vs. group RHR, *p* = 0.014, vs. group DHR, *p* = 0.047). The administration of ephedrine was significantly lower in group RHR than in group DLR (*p* = 0.045). Bradycardia was significantly lower in group DLR than in the other two groups (vs. group RHR, *p* = 0.049, vs. group DHR, *p* < 0.01). There was no significant difference in the administration of atropine among the three groups. The incidence of intraoperative hypothermia in group RHR was significantly lower than in the other two groups (vs. group DHR, *p* = 0.024; vs. group DLR, *p* < 0.01), as was the incidence of hypothermia in the PACU (vs group DHR, *p* = 0.024; vs. group DLR, *p* < 0.01). The incidence of PAS was significantly lower in group RHR than in the other two groups (vs. group DHR, *p* = 0.047, vs. group DLR, *p* = 0.02). The severity of PAS was not significantly different among the three groups (Table 1).

In terms of clinically relevant pain outcomes, the time to first rescue analgesia was significantly longer in group DLR and RHR than in group DHR (*p* < 0.01). There was no significant difference in the time to rescue analgesia between group RHR and DLR (*p* > 0.05). The total analgesic consumption (ketorolac) was not significant among the three groups. Pain intensity, as measured by a visual analog scale, was significantly higher in group DLR and RHR than in group DHR at 1 h (*p* < 0.01), 6 h (*p* < 0.01), and 12 h (*p* < 0.01) after surgery. However, there was no difference in pain intensity between group RHR and DLR. Cumulatively consumed PCA volume containing morphine for 24 h after surgery. in group DHR was significantly greater than in the other two groups (*p* < 0.01). However, there was no difference in cumulatively consumed PCA volume between group RHR and DLR (*p* > 0.05) (Table 2).

In terms of mechanically evoked pain, the preoperatively mechanical hyperalgesia threshold was not significantly different among the three groups. After 24 h of surgery, the mechanical hyperalgesia threshold was considerably lower in group DHR as compared to the other two groups (*p* < 0.01). There was no significant difference in the mechanical hyperalgesia threshold at 24 h after surgery between group RHR and DLR (*p* > 0.05). The area of hyperalgesia around the surgical incision at 24 h was postoperatively significantly greater in group DLR than in the other two groups (*p* < 0.01). However, postoperatively there was no difference in the area of hyperalgesia around the surgical incision at 24 h between group RHR and DLR (*p* > 0.05) (Table 2).

The association of PAS incidence with the post-operative mechanical hyperalgesia threshold at 24 (rpb = −0.422, *p* < 0.01), the area of post-operative hyperalgesia at 24 h (rpb = 0.454, *p* < 0.01), cumulatively consumed post-operative PCA volume containing morphine for 24 hr (rpb = 0.345, *p* < 0.01), the time to first rescue analgesia (rpb = −0.395, *p* < 0.01), the total dose of remifentanil administered (rpb = 0.375, *p* < 0.01), the incidence of intraoperative hypothermia (rφ = 0.258, *p* = 0.027), and the incidence of hypothermia in the PACU (rφ = 0.276, *p* = 0.012) (Table 3). Blood transfusion for bleeding and meperidine for the treatment of intractable PAS were not administered to the patients (data not shown).

## 4. Discussion

Based on the results of the present study, group RHR, which received remimazolam significantly attenuated mechanically evoked pain and some clinically relevant pain outcomes associated with OIH, which occurs when long-term opioid use leads to pain sensitization, compared to DHR. As for whether remimazolam attenuates or worsens pain in the present study, it showed positive results in terms of postoperative pain management. In the case of OIH, group DLR, which received a low dose of remifentanil, considered the control group in this study, exhibited better results than the group DHR, who received a high dose of remifentanil. In fact, the present study did not include a placebo control, making it impossible to determine if the observed effects were due to the anesthetic and pain management strategies or other factors [10].

The relationship between mechanically evoked pain and clinically relevant pain outcomes can be complex and multifaceted. In one study, capsaicin reduced electrical pain perception thresholds in humans and was used to map areas of hyperalgesia. This approach was utilized to rapidly assess the degree of hyperalgesia within the study population and in individual subjects [11]. However, it is important to note that these measures may not always directly correlate [12]. For example, a patient with a low threshold for mechanical hyperalgesia may not necessarily report high levels of pain on the VAS or require early rescue analgesia. Factors such as individual pain tolerance, the nature and location of the pain, concurrent medical conditions, and psychological factors can all influence these outcomes.

Indirect assessments of OIH are represented by clinically relevant pain that increases postoperative pain scores. This type of pain reduces the time to treatment with opioid analgesics and increases the dose necessary to achieve satisfactory postoperative analgesia. On the other hand, direct assessments of OIH are represented by mechanically evoked pain, such as that modeled in quantitative sensory tests with von Frey filaments. This type of pain decreases detection thresholds and increases mechanical pain sensitivity, as well as the wind-up ratio as assessed by pinprick devices and algometers. These methods are used for the direct assessment of OIH in patients under opioid-based anesthesia [13].

In some cases, there may be a strong correlation between mechanically evoked pain and clinically relevant pain outcomes. For example, a patient with a low threshold for mechanical hyperalgesia may be more likely to report high levels of pain and require early rescue analgesia [14]. However, in other cases, the correlation may be weaker or nonexistent [9]. It is important to consider all of these factors when assessing the relationship between mechanically evoked pain and clinically relevant pain outcomes. This will help to ensure that patients receive the most appropriate treatment. Therefore, while mechanically evoked pain measures such as mechanical hyperalgesia can provide valuable insights into the underlying pathophysiology of pain, they should be interpreted within the broader clinical context. It is also crucial to consider individual patient factors when assessing clinically relevant pain outcomes. In our previous single port laparoscopic gynecological surgery study in which the operation and anesthesia time were relatively short, there was no correlation between mechanically evoked pain and clinically relevant pain outcomes, but in this study, there was a moderate positive correlation [9].

Remimazolam may attenuate mechanically evoked pain and clinically relevant pain outcomes, potentially due to its effects on GABA receptors or its residual sedative properties. The effects of remimazolam on pain perception are influenced by its interaction with GABA receptors and its impact on neural processing at both spinal and supraspinal levels. Further studies are needed to clarify the effect of remimazolam on pain perception according to spinal and supraspinal levels. The residual sedative effect of remimazolam may cause patients in the RHR group to require analgesia later than in the DHR group, and sedative effects may also cause patients not to self-activate the PCA, resulting in a longer time to first rescue analgesia and a large morphine-containing PCA volume. There was no assessment of the duration required for awareness to be restored in the present study. The time to achieve full alertness after ceasing desflurane administration can vary based on factors such as the patient’s age, weight, health status, and the desflurane dose. However, in general, it takes most people about 8.2–10.3 min to fully wake up after desflurane anesthesia [15]. Full alertness is achieved after 12.3–25.0 min of cessation of the infusion of remimazolam [16].

When it comes to temperature regulation including hypothermia and PAS, remimazolam had more favorable outcomes than propofol or sevoflurane [17,18]. Group RHR had a significantly lower incidence of both intraoperative and postoperative hypothermia compared with groups DHR and DLR. The incidence of PAS was significantly higher in group DHR than in the other two groups. Previous studies [9,19,20] reported that patients who received high doses of remifentanil showed higher incidences of PAS. As a result of this study, it was found that remimazolam reduced PAS associated with hypothermia and OIH, including mechanically evoked pain and clinically relevant pain outcomes.

In adverse events, group DLR had a higher incidence of hypotension than groups RHR and DHR. They also required more ephedrine treatment. The incidence was lower in the DLR group compared to the other two groups. However, the use of atropine to manage bradycardia was similar across groups. As indicated in previous studies [21,22], remimazolam demonstrated better hemodynamic stability compared to the other two groups that received desflurane, an inhalational anesthetic.

The present study has some limitations. First, although the researchers conducted a sample size calculation, it is notable that this size was derived from a preliminary investigation. A larger sample might have allowed for a more robust analysis and the identification of more subtle differences between groups. Second, in terms of measurement techniques and outcome measurements, tools such as the VAS can be subjective, relying on the patient’s interpretation of pain. Similarly, von Frey filaments, while well-established, rely on patient feedback and can be influenced by subjective factors. The primary outcome, the mechanical hyperalgesia threshold, and several secondary outcomes such as pain intensity are inherently subjective. While they are widely used in clinical research, they are dependent on patient self-reporting and can be influenced by numerous factors. Third, the study implemented forced-air warming, when the core BT was below 36 °C. This could introduce a variable that might influence outcomes such as pain perception. Finally, while patients were given the same PCA pump setup, individual patient adherence to using the PCA device, as well as differences in postoperative care and monitoring, might introduce variability.

## 5. Conclusions

In summary, group RHR, which received remimazolam, attenuated OIH, including mechanically evoked pain and some clinically relevant pain outcomes caused by a high dose of remifentanil. However, it is necessary to take into account the residual sedative effect of remimazolam or the link between mechanically evoked pain and clinically relevant pain outcomes when interpreting the reducing effect of remimazolam on OIH. In addition, further research is essential to determine how clinically meaningful and important the small differences observed between the two groups are.

## Figures and Tables

**Table 1:** Demographic and perioperative data.

	Group RHR (*n* = 33)	Group DHR (*n* = 31)	Group DLR (*n* = 34)	*p*-Value
Age (yr)	51.0 ± 8.5	49.3 ± 6.5	49.2 ± 7.3	0.524
Sex (F/M)	11/22 (33.3/66.7)	11/20 (35.5/64.5)	14/20 (41.2/58.8)	0.789
Body weight (kg)	68.4 ± 8.4	69.0 ± 9.8	66.3± 9.6	0.470
ASA (I/II/III)	1/14/18 (3.0/42.4/54.5)	2/18/11 (6.5/58.1/35.5)	0/22/12 (3.1/55.1/41.8)	0.222
Type of surgery				0.619
Nephrectomy	11 (33.3)	8 (25.8)	10 (29.4)	
Nephroureterectomy	7 (21.2)	12 (38.7)	12 (35.3)	
Prostatectomy	15 (45.5)	11 (35.5)	12 (35.3)	
Duration of operation (min)	182.7 ± 12.3	186.1 ± 10.2	188.2 ± 11.7	0.146
Duration of anesthesia (min)	211.8 ± 12.1	214.5 ± 14.3	219.7 ± 23.1	0.535
Total fluid administered (ml)	1677.3 ± 120.3	1693.5 ± 122.3	1685.3± 130.6	0.873
Desflurane (vol%)	0	4.5 ± 0.6 *	6.0 ± 0.8	<0.01
Total dose of remifentanil administered (mg)	3.2 ± 0.6 *	3.4 ± 0.7 *	1.1 ± 0.2	<0.01
Hypotension	4 (12.1) *	5 (16.1) *	13 (38.2)	vs. Group RHR 0.014 vs. Group DHR 0.047
Bradycardia	9 (27.3) *	14 (45.2) *	3 (8.8)	vs. Group RHR 0.049 vs. Group DHR <0.01
Ephedrine	2 (6.1) *	4 (12.9)	8 (23.5)	0.045
Atropine	3 (9.1)	2 (6.5)	1 (2.9)	0.574
Intraoperative hypothermia	8 (24.2) *^†^	16 (51.6)	25 (73.5)	<0.01 * 0.024 ^†^
Hypothermia in PACU	6 (18.2) *^†^	13 (41.9)	20 (58.8)	<0.01 * 0.024 ^†^
Incidence of PAS	10 (30.3) ^†^	17 (54.8) *	9 (26.5)	0.020 * 0.047 ^†^
Severity of PAS				0.16
Grade 0	23 (69.7)	13 (41.9)	25 (73.5)	
Grade 1	3 (9.1)	4 (12.9)	3 (8.8)	
Grade 2	6 (18.2)	12 (38.7)	6 (17.6)	
Grade 3	1 (3.0)	2 (6.5)	0 (0)	

The data are presented as mean ± standard deviation or number (%). PAS; post-anesthesia shivering, * *p* vs. Group DHR. ^†^ *p* vs. Group DHR.

**Table 2:** The mechanically evoked pain and clinically relevant pain outcomes.

	Group RHR (*n* = 33)	Group DHR (*n* = 31)	Group DLR (*n* = 34)	*p*-Value
Time to first rescue analgesia (min)	25.0 ± 10.5 ^†^	10.3 ± 7.2 *	33.8 ± 10.2	0.01 * 0.04 ^†^
Total analgesic (ketorolac) consumption (mg)	38.6 ± 7.5	39.7 ± 7.3	37.5 ± 7.6	0.51
Pain intensity at movement				
VAS at 1 h	53.3 ± 8.8 ^†^	59.7 ± 8.0 *	50.5 ± 7.3	<0.01 * <0.01 ^†^
VAS at 6 h	49.8 ± 7.1 ^†^	55.0 ± 7.0 *	47.9 ± 6.3	<0.01 * <0.01 ^†^
VAS at 12 h	44.3 ± 8.8 ^†^	51.7 ± 8.4 *	41.5 ± 6.9	<0.01 * <0.01 ^†^
VAS at 24 h	36.1 ± 7.0	39.7 ± 7.6	35.5 ± 6.9	0.298
VAS at 48 h	27.2 ± 6.2	29.1 ± 7.4	25.6 ± 5.6	0.450
Cumulatively consumed PCA volume containing morphine for 24 h after surgery.	79.2 ± 10.7 ^†^	92.9 ± 9.4 *	70.1 ± 9.7	<0.01 * <0.01 ^†^
Preoperative mechanical hyperalgesia threshold at 24 h	188.0 ± 18.4	191.3 ± 19.4	184.9 ± 17.6	0.376
Mechanical hyperalgesia threshold at 24 h after surgery.	109.5 ± 28.9 ^†^	83.9 ± 23.5 *	115.1 ± 21.3	<0.01 * <0.01 ^†^
Area of the hyperalgesia at 24 h after surgery.	11.9 ± 3.1 ^†^	15.5 ± 3.4 *	10.2 ± 1.9	<0.01 * <0.01 ^†^

The data are presented as mean ± standard deviation. VAS; visual analog scale, PCA; patient control analgesia. * *p* vs. Group DLR. ^†^ *p* vs. Group DHR.

**Table 3:** The association of PAS with mechanically evoked pain, clinically relevant pain outcomes, and hypothermia.

	Mechanical Hyperalgesia Threshold at 24 h Postoperatively	Area of the Hyperalgesia at 24 h Postoperatively	PCA Volume Containing Morphine for 24 h Postoperatively	Time to First Rescue Analgesia	Total Dose of Remifentanil Administered	Intraoperative Hypothermia	Hypothermia in PACU
Incidence of PAS	*r_pb_* = −0.422	*r_pb_* = 0.454	*r_pb_* = 0.345	*r_pb_* = −0.395	*r_pb_* = 0.375	*r*_φ_ = 0.258	*r*_φ_ = 0.276
	*p* < 0.001	*p* < 0.001	*p* < 0.001	*p* < 0.001	*p* < 0.001	*p* < 0.001	*p* < 0.001

PCA; patient controlled analgesia, PACU; post-anesthesia care unit.

## Data Availability

The datasets used and/or analyzed during the current study are available from the corresponding author on reasonable request.
